# Oncology – An Oxford Core Text

**DOI:** 10.1038/sj.bjc.6600293

**Published:** 2002-05-06

**Authors:** C Gallagher

**Affiliations:** St Bartholomew's Hospital, London, UK

## Abstract

*British Journal of Cancer* (2002) **86**, 1527–1527. DOI: 10.1038/sj/bjc/6600293 www.bjcancer.com

© 2002 Cancer Research UK

## 

Edited by Roy Spence OBE and Patrick Johnston.Publisher: Oxford University Press. 2001. Paperback ISBN 0-19-262982-4 £22.95

A medium sized Oncology textbook such as this one poses the question ‘What is it for?’, since it is too big for the pocket, but necessarily abbreviated compared with the weighty comprehensive tomes. The authors state that they have primarily aimed ‘Oncology’ at undergraduate medical students, but also hope to serve nursing and para-medical staff, junior hospital doctors, general practitioners and hospice staff. However, what the undergraduate craves is simplicity and clarity, while what the practitioner encounters is the complexity of clinical diagnosis and treatment.

‘Oncology’ is arranged in a standard format with principles of cancer biology and treatment followed by particular organ system sections. Palliative and psychological care receive an early mention while oncological emergencies arrive at the end. The multiple authors around a nucleus of Belfast-based clinicians have adopted a consistent style that is clear and attractive with colour highlights to text boxes and tables. Each chapter concludes with illustrative case studies and references, although the latter suffer from textbook delay and are seldom more recent than 1998.

I found this book worked best as an undergraduate text providing a broad overview of each area. It is often clear and concise for the student audience, but rather short on the evidence for the treatments described.

Throughout the book prognostic statements are provided as summary figures for per cent 5-year or 10-year survival, which have the benefit of simplicity, but do not provide the full picture. Indeed this book must be unique amongst oncology texts in not displaying a single survival graph and in the process loses much of the information on the heterogeneity of patient and disease behaviour. There is very little discussion on how to interpret cancer trials and evaluate the evidence that they provide and no discussion about how to assess relative risk and benefit so that patients can be counselled appropriately.

Neither could I find any discussion on how to present the options and choices in a patient friendly manner that enabled the patient to participate in his or her treatment decisions, or the influence of the patient's performance status and quality of life in any of the treatment recommendations. I missed the sense of excitement that recent developments have injected into topics such as screening and genetic testing, apoptosis and chemotherapy/radiotherapy, targeted monoclonal antibody therapy, synchronous chemo and radiotherapy for squamous cancers of the head and neck, oesophageal, cervical and anal regions, or the newer opioid analgesics, such as Fentanyl in palliative care.

The classification of chemotherapeutic agents is rather idiosyncratic (e.g.; topoisomerase inhibitors), while under thoracic tumours, the aggregation of small cell lung cancers with carcinoids, while pathologically logical, made for therapeutic confusion.

Oncology has rapidly come of age in the past ten years and some of the history needs to be consigned to the archives; such as Halstead's radical mastectomy (p. 418) and the need for radiotherapists to exercise ‘full and exclusive responsibility like the surgeon’ (p. 139). The new age is complicated by the need to continually reassess complex and changing information and to include our patients in their treatment decisions. This oncology text provides a sound review for the undergraduate, but is missing much that the practitioner would find necessary for their future practice.

